# Reliability of a TMS-derived threshold matrix of corticomotor function

**DOI:** 10.1007/s00221-023-06725-3

**Published:** 2023-10-28

**Authors:** Maxine J. Shanks, John Cirillo, Cathy M. Stinear, Winston D. Byblow

**Affiliations:** 1 Department of Exercise Sciences, University of Auckland, Auckland, New Zealand; 2 Centre for Brain Research, University of Auckland, Auckland, New Zealand; 3 Department of Medicine, University of Auckland, Auckland, New Zealand

**Keywords:** Transcranial magnetic stimulation, Threshold matrix, Suprathreshold, Subthreshold, Subliminal

## Abstract

Transcranial magnetic stimulation (TMS) studies typically focus on suprathreshold motor evoked potentials (MEPs), overlooking small MEPs representing subthreshold corticomotor pathway activation. Assessing subthreshold excitability could provide insights into corticomotor pathway integrity and function, particularly in neurological conditions like stroke. The aim of the study was to examine the test–retest reliability of metrics derived from a novel compositional analysis of MEP data from older adults. The study also compared the composition between the dominant (D) and non-dominant (ND) sides and explored the association between subthreshold responses and resting motor threshold. In this proof-of-concept study, 23 healthy older adults participated in two identical experimental sessions. Stimulus–response (S–R) curves and threshold matrices were constructed using single-pulse TMS across intensities to obtain MEPs in four upper limb muscles. S–R curves had reliable slopes for every muscle (Intraclass Correlation Coefficient range = 0.58–0.88). Subliminal and suprathreshold elements of the threshold matrix showed good–excellent reliability (D subliminal ICC = 0.83; ND subliminal ICC = 0.79; D suprathreshold ICC = 0.92; ND suprathreshold ICC = 0.94). By contrast, subthreshold elements of the matrix showed poor reliability, presumably due to a floor effect (D subthreshold ICC = 0.39; ND subthreshold ICC = 0.05). No composition differences were found between D and ND sides (suprathreshold BF_01_ = 3.85; subthreshold BF_01_ = 1.68; subliminal BF_01_ = 3.49). The threshold matrix reliably assesses subliminal and suprathreshold MEPs in older adults. Further studies are warranted to evaluate the utility of compositional analyses for assessing recovery of corticomotor pathway function after neurological injury.

**Supplementary Information:**

The online version contains supplementary material available at 10.1007/s00221-023-06725-3.

## Introduction

Single pulse transcranial magnetic stimulation (TMS) can be used to obtain metrics about corticomotoneuronal (herein corticomotor, CM) pathway function and integrity in health and disease. For example, early after stroke the presence of an upper limb motor evoked potential (MEP) is indicative of a preserved CM pathway, and MEP status is a prognostic biomarker of upper limb motor recovery and outcome (Byblow et al. 2015; Stinear et al. 2017b). Beyond MEP status, MEP-derived metrics have not offered much prognostic utility even though resting motor threshold (RMT) and stimulus–response (S–R) curve slope are reliable TMS metrics for both healthy adults and sub-acute stroke patients (Carroll et al. 2001; Malcolm et al. 2006; Schambra et al. 2015). RMT reflects the ease with which CM neurons are activated at the time of stimulation (Ziemann et al. 2015). Conventionally, RMT is defined as the lowest stimulation intensity required to elicit MEPs ≥ 50 μV in at least five out of ten stimulations (Groppa et al. 2012; Rossini et al. 2015). There have been conflicting results regarding RMT differences between the dominant and non-dominant hemispheres in neurologically healthy adults. Some studies reported a lower RMT in the dominant hemisphere than in the non-dominant hemisphere (Macdonell et al. 1991; Triggs et al. 1994). However, an arguably larger body of research reports no difference between the hemispheres (Netz et al. 1995; Triggs et al. 1999; Civardi et al. 2000; Cirillo et al. 2010; Livingston et al. 2010; Shibuya et al. 2017; Souza et al. 2018). The S–R curve slope represents the gain of CM pathway excitability and is thought to reflect the progressively larger pool of CM neurons recruited with increasing stimulus intensity, the strength of the CM projections to the target muscle, and primary motor cortex glutamatergic neurotransmission (Rothwell et al. 1987; Devanne et al. 1997; Chen et al. 1998; Stagg et al. 2011; Rossini et al. 2015). Both RMT and S–R curve slope are useful metrics to identify and characterise pathophysiology, and aid in the diagnosis of various neurological conditions (Vucic et al. 2023).

Whilst RMT and S–R slopes are useful TMS parameters in research and clinical environments, less attention has been given to small MEPs with amplitudes that fall below the 50 μV criterion for RMT. Such subthreshold MEPs may provide valuable information about the integrity and function of the CM pathway, particularly in the presence of neurodegenerative disorders such as multiple sclerosis and Parkinson’s disease, and early after neurological injury such as stroke (Byblow et al. 2015; Schambra et al. 2015; Cirillo et al. 2020; Vucic et al. 2023). For example, characterising subthreshold MEPs may improve prediction accuracy of upper limb motor outcome after stroke. The present study explores a new approach for examining peri-threshold activation of the CM pathway using TMS-derived MEPs in healthy older adults in the first instance. TMS was delivered at a range of stimulus intensities and responses were recorded from four upper limb muscles. A novel threshold matrix was used to combine for analysis suprathreshold and subthreshold MEPs, as well as stimulus intensities that produced no MEPs.

Suprathreshold elements of the threshold matrix are the combinations of muscles and stimulation intensities that yield MEPs which meet the conventional criterion for RMT. Subthreshold elements are the combinations of muscles and intensities that produce MEPs below the RMT criterion. The subliminal elements of the threshold matrix are the combinations of muscles and intensities with no detectable MEPs. The proportion of each of the three elements making up the threshold matrix can then be calculated. We propose that this new threshold matrix may offer a better understanding of early neurophysiological recovery after stroke affecting the motor system.

It is informative and necessary to first determine the reliability of the novel metrics that can be derived from a threshold matrix (De Vet et al. 2011). Therefore, the aim of the present study was to examine the test–retest reliability of metrics derived from a threshold matrix compositional analysis in neurologically healthy older adults. Threshold matrices were constructed using MEP data obtained to produce conventional S–R curves. The reliability of the novel composite measure, S–R curve slopes, and RMT was determined through a typical test–retest procedure with experimental sessions one week apart. Additionally, threshold matrix elements from the dominant and non-dominant sides were compared, and the relationship between the subthreshold element and RMT was explored. The primary hypothesis was the threshold matrix elements would be reliable for both dominant and non-dominant sides. Secondary analyses also allowed us to determine differences in the threshold matrix elements between the dominant and non-dominant sides, distal and proximal muscles, and if there was an association between the subthreshold element of the threshold matrix and RMT. Reliability data for threshold matrix compositional analysis will inform the suitability of this approach for quantifying early post-stroke recovery, particularly at the time when subthreshold MEPs are often observed.

## Materials and methods

### Participants

Twenty-three healthy older adults volunteered to take part in the experiment. Volunteers were included if they were 50–90 years old, self-reported to have no neurological conditions, and had no contraindications to TMS assessed using a safety checklist. Participants’ handedness was assessed with the short version of the Edinburgh Handedness Inventory (Veale 2014). Written informed consent was obtained before participation. The study was approved by the Auckland Health Research Ethics Committee in accordance with the declaration of Helsinki (REF AH24292).

### Experimental arrangements

Two identical experimental sessions were used to investigate the test–retest reliability of the neurophysiological measures. The two sessions were completed at the same time of day, one week apart. For two participants, the sessions were three months apart due to equipment failure. Each session lasted approximately 2 h. The same experimenter responsible for data collection (MJS) completed all sessions which were conducted in the same laboratory at the University of Auckland.

Participants were seated comfortably in a chair with their arms resting on a pillow on their lap or hanging at their side. The position of the arms was chosen based on participant preference and optimal relaxation, which was determined by visual inspection of electromyography (EMG) recordings. The arm position for each participant was kept consistent across sessions.

Frameless stereotaxic neuronavigation (Brainsight^®^, Rogue Research Inc., Montreal, Canada; Polaris Vicra^®^, Northern Digital Inc., Ontario, Canada) was used to ensure consistent stimulation sites within and between experimental sessions. Briefly, the infrared camera tracked the participant via a reflective marker set attached near the centre of the forehead and secured with an elastic headband. A reflective marker set was also positioned on the handle of the TMS coil. The participant’s head position was co-registered to a template brain (MNI ICBM 152 average brain). The registration process was refined until the error was less than 3 mm.

#### Electromyography recordings

Surface EMG was recorded bilaterally from the *first dorsal interosseous* (FDI), *abductor digiti minimi* (ADM), *extensor carpi radialis* (ECR), and *flexor carpi radialis* (FCR) muscles. Muscle activity was recorded using 25-mm-diameter Ag–AgCl surface electrodes (Cleartrode™ RTL, ConMed, USA) arranged using a consistent belly-tendon montage appropriate for each muscle. A common ground electrode was placed on the dorsum of the left hand. The EMG signals were amplified (× 1000), band-pass filtered (10–1000 Hz), and sampled at 2000 Hz with a CED interface system (POWER1401mkll; Cambridge Electronic Design, Cambridge, UK). EMG data were recorded for 1 s, including a 0.5 s pre-stimulus window. Rectified and smoothed pre-stimulus EMG data were visually displayed to the participant, with a target line at 10 μV overlaid to assist maintenance of a resting state. Participants were instructed to keep the rectified, smoothed EMG trace for all muscles below the target line. Data were saved to a computer for offline analysis using Signal software (version 7.07, Cambridge Electronic Design).

#### Transcranial magnetic stimulation

TMS was applied using a figure-of-eight coil (MC-B70) connected to a MagPro X100 stimulator with Option, used in Power mode (MagVenture, Farum, Denmark). Due to technical complications, a MagStim 200^2^ stimulator with a D70^2^ coil (The Magstim Company Ltd., Whitland) was used with four participants. For each participant, the same stimulator was always used for both sessions. The coil was held over the M1 area with the handle posterolateral at approximately 45° from the midline. Monophasic stimulation was used to induce a posterior-to-anterior current in the brain.

An optimal position for eliciting MEPs in all four contralateral muscles was determined and recorded using the Brainsight^®^ software. This ‘global’ hotspot was used to elicit MEPs in all four upper limb muscles. The optimal coil position for eliciting MEPs in the contralateral FDI was also assessed and marked with Brainsight^®^ as the ‘FDI’ hotspot. All recorded hotspots were stored and used in the second session to ensure the same stimulation site was used in both sessions. Sites were confirmed physiologically based on MEP presence.

A maximum-likelihood parameter estimation by sequential testing strategy without a priori information was used to determine FDI RMT (Awiszus and Borckardt 2011) as the lowest stimulator output required to elicit MEPs ≥ 50 μV in 50% of trials (Rossini et al. 2015). Separate FDI RMT values were obtained from stimulation at the global and FDI hotspots. For all participants, the right hemisphere global hotspot was investigated first, followed by the right hemisphere FDI hotspot, before completing the same process for the left hemisphere. A fixed testing order was proposed in a previous reliability study to ensure that any potential order effects consistently influence measurement variability (Schambra et al. 2015).

For the construction the threshold matrix, ten stimuli at ten intensities were delivered to the global hotspot. Stimulation intensities were fixed and ranged from 10 to 100% maximum stimulator output (MSO) in 10% increments. For the construction of S–R curves, ten stimuli at 11 intensities were used with 65% MSO as the additional intensity. The 65% MSO intensity was included to increase sensitivity around the midpoint of the linear portion of the S–R curve. Stimulation intensities were randomised and delivered at an inter-stimulus interval of 6 s with 15% variability.

### Data analysis and statistics

Data were processed using custom scripts in MATLAB (R2020b, v9.9; The MathWorks). All statistical analyses were performed in JASP (JASP Team (2022). JASP (Version 0.16.4)).

### Threshold matrix construction

A time–frequency analysis of the EMG data was applied to identify the presence or absence of MEPs, similar to a previous report (Tecuapetla-Trejo et al. 2021). This method of automatic MEP detection has a similar performance to manual inspection of EMG traces whilst providing time saving benefits and decreasing manual inspection subjectivity (Tecuapetla-Trejo et al. 2021). Trials with a root mean square (RMS) greater than 15 μV in a 50 ms pre-stimulus window were excluded from analysis for each muscle.

A Short-Time Fourier Transform (STFT) was applied to each EMG signal from each trial using a window size of 10 ms with a 5 ms overlap. The STFT outputs are a frequency vector, a time vector, and a matrix with complex STFT coefficients across both time and frequency. The frequencies ranged from 0 to 1000 Hz. The power spectral density (PSD) was calculated by squaring the absolute value of the STFT coefficients. The PSD represents the distribution of the signal’s energy into frequency components (Dempster 2001). The maximum PSD value was determined for each time window by identifying the highest power observed across all frequency ranges. Across all trials for each muscle, the largest maximum PSD value in a 50 ms pre-stimulus time window was identified and set as the criterion for MEP detection. If the maximum PSD value in a 15–30 ms post-stimulus window was greater than the criterion set from the pre-stimulus PSD values, the trial was deemed to have a MEP response. For trials with a MEP, the peak-to-peak MEP amplitude and pre-stimulus RMS were calculated from the same time windows used for the PSD analysis.

The threshold matrix has forty elements comprised of ten stimulation intensities and four muscles. Stimulation intensities ranged between 10 and 100% MSO in 10% increments. Ten stimuli were delivered at each intensity. If trials were rejected due to noise or background EMG, at least five trials had to be retained for any given intensity and muscle. If less than five trials were available for analysis for any muscle and intensity, the participant’s data were excluded from the analysis.

Each cell of the threshold matrix was colour-coded based on RMT criteria. Stimulation intensity and muscle combinations that resulted in MEPs ≥ 50 μV in at least 50% of trials were coloured green. Stimulation intensity and muscle combinations that produced MEPs which did not meet RMT criteria were coloured orange. Stimulation intensity and muscle combinations that did not produce any MEPs were coloured red.

**Fig. 1:**
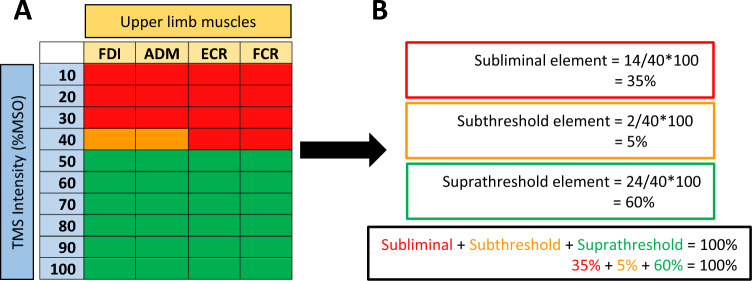
Threshold matrix construction. **A** A 40-cell threshold matrix comprised of four upper limb muscles (columns) and TMS intensity in %MSO (rows). Other variations are possible. Cells that produce a set of MEPs that meet the criteria for RMT are coloured green. Cells that produce a set of MEPs that fail to meet RMT criteria are coloured orange. Cells that do not elicit MEPs are coloured red. **B** Threshold matrix composition is calculated by determining the proportion of each coloured element. The red, orange, and green elements are termed the subliminal, subthreshold, and suprathreshold elements, respectively and sum to 100%. *FDI* first dorsal interosseous, *ADM* abductor digiti minimi, *ECR* extensor carpi radialis, *FCR* flexor carpi radialis

A schematic of threshold matrix construction using a participant’s data is shown in Fig. 1. Threshold matrix composition was calculated across the four muscles and ten stimulation intensities by dividing the number of cells of the colour of interest by the total number of squares in the matrix (i.e. 40) and multiplying by 100. The green, orange, and red compositional elements are termed suprathreshold, subthreshold, and subliminal, respectively.

### Compositional data analysis

The three compositional elements in the matrix are constrained to sum to 100% and any change in one matrix element will necessarily change one or both remaining elements. This means the relationship between the elements is of interest, rather than the absolute sum (Greenacre 2021). Elements cannot have negative values as they are proportions. Together with the constant-sum constraint, this means that the data do not fit the assumptions of normality and must be transformed before applying parametric statistical techniques. Logarithms of ratio transformations (log ratios) were applied to the data, preserving the elements’ composition but removing the dependency between elements (Greenacre 2021). Amalgamation log ratios (ALRs) are used as they are more readily interpreted than balance measures obtained from isometric log ratios (Greenacre et al. 2021). Each ALR was computed as one element relative to the sum of the remaining elements. For example, the ALR for the suprathreshold element was calculated as ln(suprathreshold/(subthreshold + subliminal)). Calculating the exponent of the ALR (eALR) returns the percentage difference of the numerator compared to the denominator (Greenacre et al. 2021). Zero values can become problematic in compositional data analysis due to their incompatibility with log ratios. As such, a conventional replacement method has been used in this dataset, whereby the zeros were replaced with 65% of the smallest unit, which is one cell of the threshold matrix (i.e. 0.65*(1/40*100)) (Martín-Fernández et al. 2003). Once the ALRs were calculated, parametric statistics were conducted.

The average threshold matrix composition was calculated across the two sessions for each participant from the dominant and non-dominant sides. The average compositions from the dominant and non-dominant sides were visualised in a ternary plot along with the centre of the composition, calculated using geometric means. Ternary plots are the standard graphical tool for visualising three-part compositional data sets with each vertex representing one element as a percentage. The sum of the three percentages for any data point always equal 100%. The closer a data point is to any vertex, the more of that element is present in the composition.

### Stimulus–response curve construction

The pre-stimulus window was set for 50 ms prior to stimulation onset. Trials were removed if pre-stimulus EMG RMS was greater than 15 μV. The MEP amplitude window width was 50 ms, starting 10 ms post stimulation onset. Averages of pre-stimulus RMS and MEP amplitude for each stimulation intensity for each muscle were calculated. The average MEP amplitude for each muscle was plotted as a function of TMS intensity, and a sigmoid function was fitted in MATLAB, similar to previous studies (Capaday 1997; Devanne et al. 1997). The slope of the function at S50 was determined as a measure of the gain of the corticomotor pathway (Devanne et al. 1997). The S–R curve slopes are expressed in mV/10% MSO.

### Reliability measures

Intraclass correlation coefficients (ICC) were used to examine test–retest reliability (De Vet et al. 2011). The ICC indicates the extent to which people in the sample could be distinguished from one another in the presence of measurement error (Polit 2014). ICC scores range from 0 to 1, with higher scores representing less measurement error and higher reliability. The following criteria are commonly used for ICC interpretation: < 0.5 poor reliability; 0.5 ≥ 0.75 moderate reliability; 0.75 ≥ 0.9 good reliability; > 0.9 excellent reliability (Koo and Li 2016).

Before performing the ICC calculations, the normality of the S–R curve slope and RMT data were checked using Shapiro–Wilk tests and visual inspection of Q–Q plots. Logarithmic transformations were used to correct non-normal data.

The ICC was calculated for the three elements of the threshold matrix, RMT from both the global and FDI hotspot, and the slope of the S–R curve obtained for each muscle. All ICC calculations were determined from data obtained for both the dominant and the non-dominant sides. An ICC_3,1_ model was used to fit the data obtained from the two-way mixed effects and single rater design. Bland–Altman plots were used to visually inspect the test–retest measures (see supplementary material).

### Dominant versus non-dominant sides

Bayesian paired t-tests were used to assess differences in the threshold matrix elements between the dominant and non-dominant sides. The ALRs were calculated from the geometric mean composition across the two sessions for each participant. The Bayes Factor in favour of the null hypothesis (BF_01_) was calculated with Bayesian paired *t *tests. A BF_01_ greater than one supports the null hypothesis and values less than one support the alternative hypothesis. The strength of evidence for the null hypothesis was determined by interpreting the effect size as small (BF_01_: 1–3), medium (BF_01_: 3–10), or large (BF_01_: > 10) (van Doorn et al. 2021).

### Subthreshold responses versus resting motor threshold correlations

A Bayesian correlational analysis was performed for both the dominant and non-dominant sides to assess whether the subthreshold element of the threshold matrix was associated with RMT. Support for the null hypothesis was determined by the BF_01_ value using the same criterion as above.

### Threshold matrix composition of distal versus proximal muscles

Bayesian paired *t* tests were used to compare the threshold matrix composition of the two hand and two forearm muscles. The dominant and non-dominant threshold matrices for each participant were split into an FDI and ADM, and an ECR and FCR matrix. The same compositional data analysis process was used with the two muscle matrices as the four muscle matrices. The average hand and forearm composition was calculated for each participant across the two sessions. Support for the null hypothesis was determined by the BF_01_ value using the same criterion as above.

### Resting motor threshold comparisons

Bayesian paired t-tests were used to assess differences in the RMT values obtained from the global and FDI hotspot for the dominant and non-dominant sides. The average RMT value was calculated for each participant across the two sessions. Support for the null hypothesis was determined by the BF_01_ value using the same criterion as above.

## Results

All participants completed both experimental sessions (*N* = 23, 13 females, 10 males; mean age: 67.7 years; range 51–87 years). Twenty-one participants were right-handed (mean laterality quotient 0.94, range 0.25–1), and two were left-handed (mean laterality quotient − 0.80, range − 1 to − 0.60). No adverse events were reported.

The average Brainsight^®^ registration error was 1.3 mm (0–2.8 mm). The TMS coil position relative to the hotspot target was visually monitored throughout the session. Across all targets, the coil was positioned on average 0.7 mm away from the target with 2.2° of tilt and 0.2° of rotation, respectively.

The average RMT for the dominant side was 41 ± 10% MSO from the global hotspot and 39 ± 9% MSO from the FDI hotspot. The average RMT for the non-dominant side was 41 ± 8% MSO and 40 ± 7% MSO from the global and FDI hotspot, respectively. Due to a recording error, RMT was not obtained for one participant. A summary of participant demographic and neurophysiological characteristics is provided in Table 1.

**Table 1:** Demographic and neurophysiological characteristics

Side	Dominant	Non-dominant
**FDI RMT (%MSO)**	*N* = 22	*N* = 22
Global hotspot	41 ± 10 (range 26–63)	41 ± 8 (range 25–59)
FDI hotspot	39 ± 9 (range 26–62)	40 ± 7 (range 26–61)
**S–R curve slopes (mV/10%MSO)**	*N* = 22	*N* = 22
FDI	2.27 ± 2.52	2.05 ± 1.66
ADM	2.16 ± 1.87	1.54 ± 1.18
ECR	0.80 ± 0.80	0.74 ± 0.50
FCR	0.75 ± 0.66	0.58 ± 0.53
**Threshold matrix element ALR**	*N* = 21	*N* = 18
Suprathreshold	0.46 ± 0.47	0.55 ± 0.35
Subthreshold	− 2.64 ± 0.55	− 2.90 ± 0.50
Subliminal	− 0.75 ± 0.44	− 0.79 ± 0.34

Age, Edinburgh Handedness Score, RMT, S–R curve slope, and ALR measures are mean ± standard deviation

*RMT* resting motor threshold, *S–R* stimulus–response, *FDI* first dorsal interosseous, *ADM* abductor digiti minimi, *ECR* extensor carpi radialis, *FCR* flexor carpi radialis, *ALR* amalgamation log ratio

**Fig. 2:**
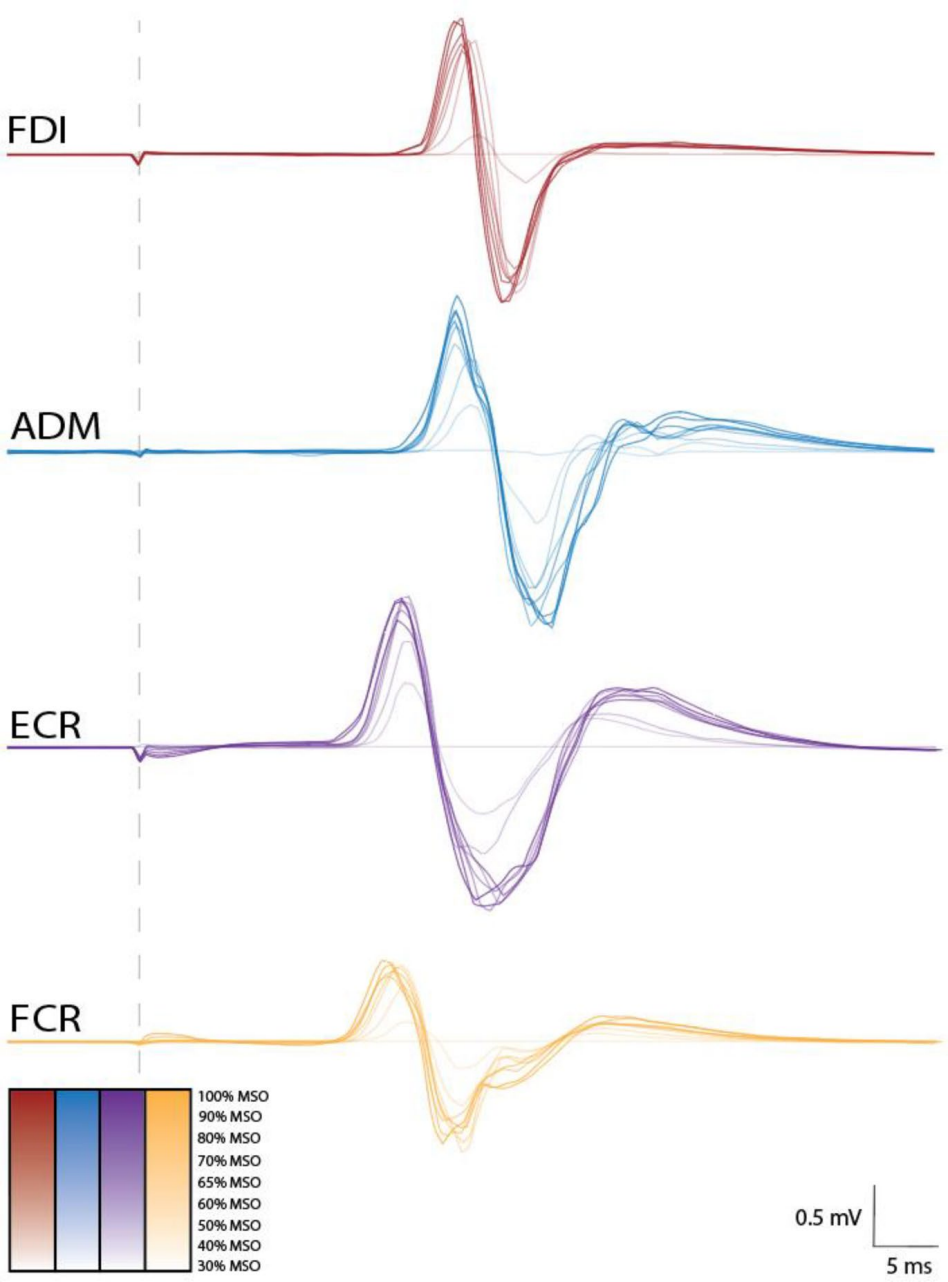
Electromyography traces. Electromyography (EMG) traces from the FDI, ADM, ECR, and FCR muscles. Each trace is the average of ten trials at intensities ranging from 30 to 100% MSO for one participant. A colour legend for stimulus intensity is displayed. Most, but not all traces contain a motor evoked potential (MEP). The vertical dashed line represents time of stimulation. Calibration bars for amplitude and time are displayed. *FDI* first dorsal interosseous, *ADM* abductor digiti minimi, *ECR* extensor carpi radialis, *FCR* flexor carpi radialis

**Fig. 3:**
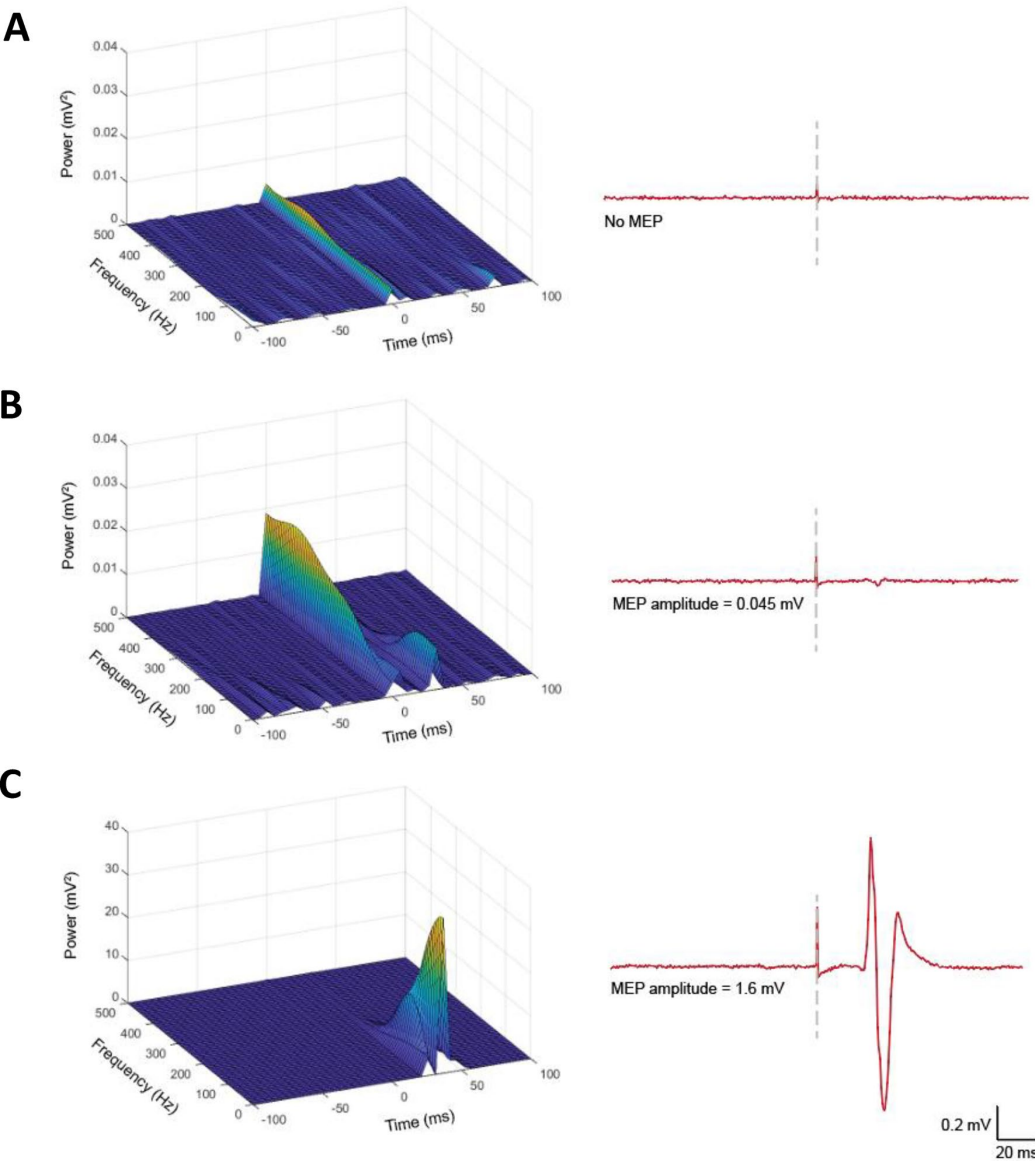
Time–frequency visualisation of traces with no (**A**), a subthreshold (**B**) and suprathreshold (**C**) motor evoked potential. Left hand panel contains time–frequency visualisations of the time-domain traces in the right hand panel. Note the power scale difference between panels **A**, **B** and **C**. The colour scale is associated with the z-axis, with warmer colours representing a stronger signal. The dashed lines on the time-domain traces in the right hand panel indicate the stimulus artefact. In panels **A** and **B**, the stimulus artefact is visible; however, it is not discernible in panel **C** due to the scale required to display the suprathreshold MEP power. Calibration bars show amplitude and time for the time-domain traces

**Fig. 4:**
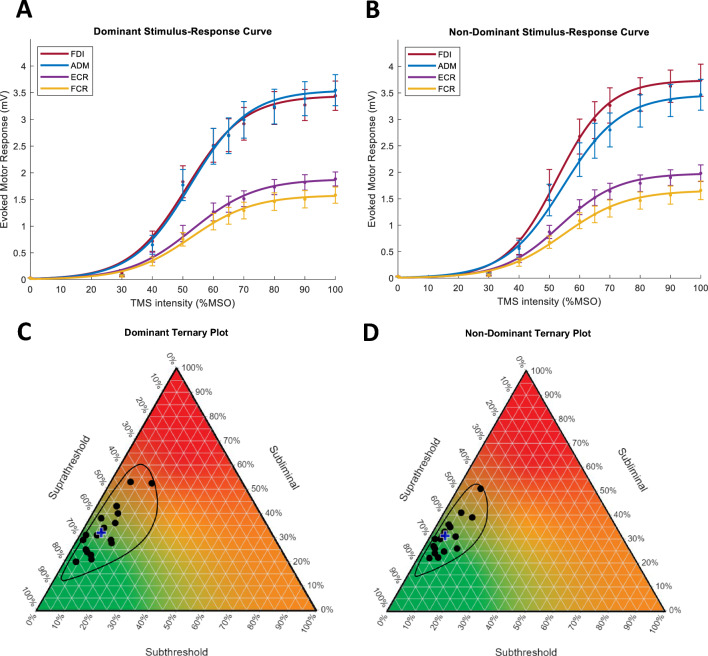
Stimulus–response curves and ternary plots from the dominant (**A** and **C**) and non-dominant (**B** and **D**) sides. **A**, **B** S–R curves show evoked motor response amplitude (mV) as a function of TMS intensity (%MSO). Each data point is an average of 22 participants. Error bars represent standard error of the mean. Ternary plots illustrate the threshold matrices from the dominant side for 21 participants (**C**) and from the non-dominant side for 18 participants (**D**). Each black circle represents the average composition across the two experimental sessions for one participant. Some threshold matrices have the same composition, therefore overlaid. Each vertex represents one element of the composition as illustrated with the colour map. The blue cross represents the centre of the compositions calculated using the geometric mean. The three-part composition can be read using the gridlines associated with each axis of the triangle. *FDI* first dorsal interosseous, *ADM* abductor digiti minimi, *ECR* extensor carpi radialis, *FCR* flexor carpi radialis

Example EMG traces are displayed in Fig. 2. Visualisation of the time-frequency analysis for example EMG traces are displayed in Fig 3. S–R curves could be constructed for 22 participants. Plots of the average S–R curves across the two sessions for both the dominant and non-dominant sides are displayed in Fig. 4A, B. After pre-stimulus RMS and the minimum number of trials criteria were applied, threshold matrices could be constructed for 21 participants for the dominant side and 18 participants for the non-dominant side. The average number of retained trials per stimulation intensity were 9.8, 9.7, 9.6, and 9.7 for the dominant FDI, ADM, ECR, and FCR, respectively. The average number of retained trials per stimulation intensity for the non-dominant FDI, ADM, ECR and FCR were 9.7, 9.9, 9.8, and 9.7, respectively. No MEPs were observed with 10% MSO or 20% MSO stimulation intensities. Threshold matrices for both the dominant and non-dominant sides are displayed in ternary plots in Fig. 4C, D.

### Reliability measures: intraclass correlation coefficients

The ICC and the associated 95% lower and upper confidence intervals for RMT values, threshold matrix elements, and S–R curve slopes for the dominant and non-dominant sides are provided in Table 2.

**Table 2:** Reliability analysis from intraclass correlation coefficients for dominant and non-dominant sides

Side	Dominant	Non-dominant
**Resting motor threshold**	*N* = 22	*N* = 22
Global	0.90 (0.78, 0.96)	0.86 (0.70, 0.94)
FDI	0.95 (0.89, 0.98)	0.81 (0.62, 0.91)
**Threshold matrix elements**	*N* = 21	*N* = 18
Suprathreshold	0.92 (0.82, 0.97)	0.94 (0.85, 0.98)
Subthreshold	0.39 (− 0.04, 0.70)	0.05 (− 0.42, 0.49)
Subliminal	0.83 (0.63, 0.93)	0.79 (0.52, 0.92)
**S–R curve slopes**	*N* = 22	*N* = 22
FDI	0.88 (0.73, 0.95)	0.58 (0.22, 0.80)
ADM	0.87 (0.72, 0.95)	0.80 (0.57, 0.91)
ECR	0.81 (0.59, 0.91)	0.73 (0.45, 0.88)
FCR	0.78 (0.53, 0.90)	0.88 (0.74, 0.95)

ICC with lower and upper 95% confidence intervals in parentheses. An ICC of > 0.75 is considered good reliability

*FDI* first dorsal interosseous, *ADM* abductor digiti minimi, *ECR* extensor carpi radialis, *FCR* flexor carpi radialis, *S–R* stimulus–response

#### Resting motor threshold

RMT values from the global and FDI hotspot for the non-dominant side showed good reliability at 0.86 and 0.81, respectively. For the dominant side, RMT values obtained from the global and FDI hotspot demonstrated excellent reliability at 0.90 and 0.95, respectively.

#### Threshold matrix elements

For both the dominant and non-dominant sides, the suprathreshold element of the threshold matrix had excellent reliability, with ICC values of 0.92 and 0.94, respectively. The subliminal element of the threshold matrix had good reliability with an ICC value of 0.83 from the dominant side and 0.79 from the non-dominant side. The subthreshold element of the threshold matrix had poor reliability for both sides. The dominant and non-dominant ICC values were 0.39 and 0.05, respectively. The poor reliability was not unexpected, given that few, if any, subthreshold MEPs can be obtained from healthy participants.

#### Stimulus–response curve slopes

The S–R curve slopes obtained from the dominant side for FDI, ADM, ECR, and FCR muscles showed good reliability with ICC values of 0.88, 0.87, 0.81, and 0.78, respectively. For the non-dominant side, S–R curve slopes for ADM and FCR showed good reliability with ICC values of 0.80 and 0.88, respectively. The S–R curve slope for the non-dominant FDI and ECR had moderate reliability with ICC values of 0.58 and 0.73, respectively.

### Dominant versus non-dominant sides

Eighteen participants were included in the analysis of the threshold matrices obtained from the dominant and non-dominant sides. There were no significant differences in any of the threshold matrix elements between the dominant and non-dominant sides. A moderate effect size supported the null hypothesis of no difference in suprathreshold and subliminal elements of the threshold matrix between dominant and non-dominant sides (suprathreshold BF_01_ = 3.85, 95% Credible Interval: − 0.35, 0.51; subliminal BF_01_ = 3.49, 95% Credible Interval: − 0.30, 0.56). There was a small effect size when comparing the dominant and non-dominant subthreshold elements (BF_01_ = 1.68, 95% Credible Interval: − 0.75, 0.14).

### Correlation analyses

For the dominant side, 20 participants were included in the correlational analysis. There was no relationship between subthreshold responses and RMT from the dominant side (*r* = 0.41, BF_01_ = 0.82). For the non-dominant side, seventeen participants were included in the correlational analysis. There was no relationship between the subthreshold element and RMT (*r* = 0.32, BF_01_ = 1.60).

### Threshold matrix composition of distal versus proximal muscles

For the dominant side, 21 participants were included in the Bayesian paired t-test analysis. There were no differences in any threshold matrix element between the distal and proximal muscle groups (suprathreshold BF_01_ = 3.22, 95% Credible Interval: − 0.24, 0.57; subthreshold BF_01_ = 4.25, 95% Credible Interval: − 0.35, 0.45; subliminal BF_01_ = 2.67, 95% Credible Interval: − 0.62, 0.20). For the non-dominant side, 18 participants were included in the Bayesian paired t-test analysis. There were no differences in any threshold matrix element between the distal and proximal muscle groups (suprathreshold BF_01_ = 3.55, 95% Credible Interval: − 0.55, 0.32; subthreshold BF_01_ = 0.62, 95% Credible Interval: − 0.93, 0.01; subliminal BF_01_ = 2.58, 95% Credible Interval: − 0.22, 0.65).

### Resting motor threshold comparisons

Twenty-two participants were included in the analysis of RMT between the global and FDI hotspots for both the dominant and non-dominant sides. There were no differences between RMT values obtained from the global and FDI hotspots for the dominant or non-dominant sides. For the non-dominant side, a moderate effect size supported the null hypothesis of no difference in RMT between the global and FDI hotspots (BF_01_ = 3.05, 95% Credible Interval: − 0.22, 0.58). For the dominant side, the BF_01_ factor is small and approximating 1 (BF_01_ = 0.60, 95% Credible Interval: 0.00–0.86). This finding can be interpreted as the RMT from the global and FDI hotspots are neither the same nor significantly different.

## Discussion

Threshold matrices were successfully constructed for a cohort of neurologically healthy older adults. The suprathreshold and subliminal elements of the threshold matrix were reliable in this cohort. The subthreshold element had poor reliability, and this is most likely due to the very low number of subthreshold responses obtained from these neurologically healthy participants. There were no differences in threshold matrix composition between dominant and non-dominant sides and no association between subthreshold responses and RMT. These findings support the robustness of the threshold matrix composition as a reliable metric irrespective of a person’s RMT or which hemisphere it is obtained from.

### Subthreshold element

The poor reliability of the subthreshold element is presumably driven by its relatively small contribution to the threshold matrix composition of healthy adults. An infrequently and minimally present element cannot be expected to demonstrate good reliability. The lack of subthreshold excitability becomes apparent when comparing the boundaries between threshold matrix elements and S–R curve parameters. The far-left portion of the S–R curve represents subliminal stimulus intensities. There are no MEPs at these TMS intensities, therefore, the y-axis MEP amplitude value is zero. Once RMT intensity is reached, the foot of the S–R curve deviates rapidly from zero, entering its linear portion. In the absence of nervous system pathology, the transition from no MEPs (i.e. 0 mV) to persistent MEPs ≥ 50 μV occurs expeditiously. This transition at the foot of the S–R curve is equivalent to the boundary between the subliminal and suprathreshold elements of the threshold matrix. Therefore, subthreshold responses were very infrequent, and the subthreshold element was relatively small and variable in the threshold matrices constructed. Reliability measures are specific to the population assessed. Whilst the subthreshold element had poor reliability in the present study, this would not necessarily be the case in a cohort of patients early after stroke and requires further testing.

### Hypothesis generation for a stroke cohort

The present study is a necessary starting point for establishing the threshold matrix composition of healthy adults. Deviations from compositions seen in this study could provide interesting neurophysiological insights for stroke recovery. For example, S–R curves have a shallower slope and a lower maximal amplitude plateau in stroke patients (Liepert et al. 2005; Swayne et al. 2008; Schambra et al. 2015; Stinear et al. 2015). The S–R curve post-stroke is characterised by a more gradual increase in MEP amplitude at the foot of the S–R curve. It is reasonable to expect that more subthreshold responses would be present in the threshold matrix of sub-acute stroke patients, when spontaneous biological recovery processes are occurring (Zeiler and Krakauer 2013; Stinear and Byblow 2014; Krakauer and Carmichael 2017). A greater prevalence of subthreshold responses in stroke patients could reflect fewer CM neurons, the desynchronised firing of the remaining neurons, and demyelination and remyelination processes.

Investigating matrix composition early after stroke may provide new prognostic information about the integrity and function of the CM pathway compared to a binary MEP status measure (Stinear et al. 2012, 2017b; Byblow et al. 2015). MEP status is the key biomarker used in the PREP2 prediction tool which is currently the most accurate, externally validated tool for predicting upper limb functional outcome post-stroke (Stinear et al. 2017b). PREP2 uses a combination of clinical measures and MEP status within the first week after stroke to predict a patient’s upper limb outcome at three months. Predicted outcomes fall into one of four categories: Excellent, Good, Limited, or Poor. Each category is defined by a range of Action Research Arm Test scores three months after stroke. All patients who are MEP positive (MEP+) with TMS are predicted to have a Good outcome whereas MEP negative (MEP-) patients are given a Limited or Poor prediction for their upper limb depending on their stroke severity. However, not all MEP+ patients achieve a Good upper limb outcome within three months after stroke. (Stinear et al. 2012, 2017b).

**Fig. 5:**
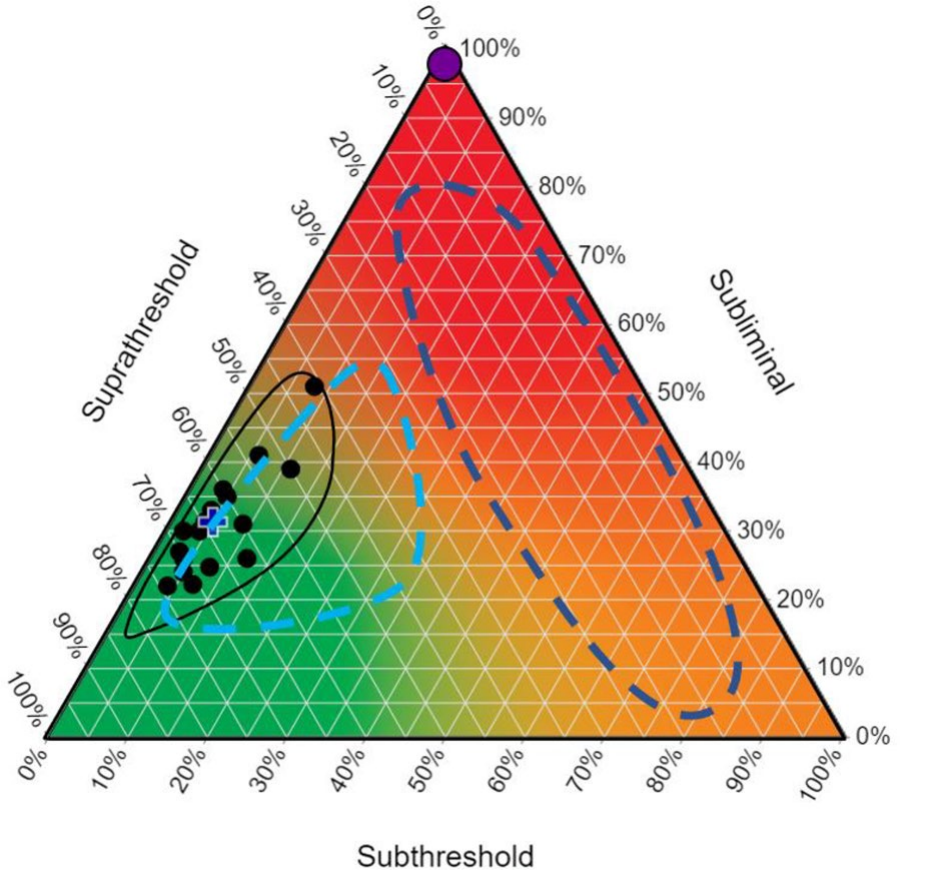
Hypothetical regions of threshold matrix composition for patients with and without a functional corticomotor pathway after stroke. Hypothetical predictive regions for threshold matrix composition of stroke patients are overlaid on the non-dominant ternary plot in neurologically healthy older adults (Fig. 4D). The solid purple ellipse represents the 100% subliminal threshold matrix of MEP- patients, without a viable CM pathway. The light blue dashed ellipse is the hypothesised region of the threshold matrices for MEP+ patients who may achieve or exceed their predicted Good upper limb outcome. The dark blue dashed ellipse is the hypothesised region of the threshold matrices for MEP+ patients who may not achieve their predicted Good upper limb outcome. Both the light and dark blue groups together would be considered MEP+ with a viable CM pathway (Stinear et al. 2012; Stinear et al. 2017a, b). The extent of non-overlapping regions for MEP + patients may help distinguish between recovery phenotypes

Figure 5 conceptualises hypothesised threshold matrix compositions for the lesioned hemisphere of stroke patients with upper limb involvement, overlaid on the ternary plot of the data from the current study. In Fig. 5 three hypothesised predictive regions are categorised by MEP status and upper limb outcome (Byblow et al. 2015; Stinear et al. 2017b). MEP- patients sit at the subliminal vertex, as they have a 100% subliminal matrix composition. For a patient to be classified as MEP+ , a minimum of one cell of subthreshold excitability is needed (Stinear et al. 2017a). However, the threshold matrices of MEP+ patients could range from one cell of subthreshold excitability to many cells resembling the matrices of neurologically healthy older adults, or anywhere in between. This range of possible threshold matrices may carry important predictive information that is not captured by the binary MEP status biomarker.

It is possible that the variability in the upper limb outcome achieved by MEP+ patients is influenced by neurobiological factors that may only be evident in measures that are sensitive to subthreshold excitability early after stroke. In Fig. 5, we hypothesise that stroke patients with a threshold matrix resembling healthy adults are more likely to achieve or exceed their predicted Good outcome. Conversely, patients with threshold matrices that appear higher on the subliminal and subthreshold axes may not achieve a Good outcome. If true, the threshold matrix may be able to more accurately predict upper limb outcome for MEP+ patients than binary MEP status. These ideas, whilst speculative, seem biologically plausible and require evaluation with a patient cohort.

### Global hotspot

In TMS neurophysiology experiments an optimal stimulation site is usually determined for a specific muscle of interest. Upper limb musculature has a somatotopic gradient in the primary motor cortex, meaning the optimal stimulation site for distal musculature is located more laterally than proximal muscles (Penfield and Boldrey 1937). However, cortical mapping studies have shown distal muscle representations in the primary motor cortex overlap considerably, with no significant differences between hotspot locations (Wassermann et al. 1992; Devanne et al. 2006). Furthermore, increasing stimulus intensity at a given stimulation site results in activation of muscle representations at greater distances from the stimulation site. Most recent evidence points towards a hand region in the primary motor cortex with a concentric organisation, that is, fingers as the most distal part represented in the centre, with proximal shoulder areas at the edges of the hand region (Gordon et al. 2022). In the present study, there was no difference between the FDI RMT value obtained from the global hotspot and the FDI specific hotspot from either the dominant or non-dominant side. Additionally, RMT values from both hotspots displayed good or excellent reliability. Therefore, using a single hotspot to evaluate multiple closely located muscles offers practical and time-saving advantages whilst obtaining the data required for S–R curve and threshold matrix construction, which might be particularly relevant for patient studies.

### Time–frequency analysis

MEP analyses are typically restricted to the time domain and capture changes in the EMG voltage signal over time (peak-to-peak amplitude or latency). However, these measurements can be sensitive to noise from varying sources (Merletti and Farina 2016), and affected by temporal dispersion of the descending CM volleys resulting in polyphasic MEPs (Rossini et al. 2015). When averaging EMG signal features in the time domain positive and negative deflections cancel each other out. If the signal feature is not phase-locked, which is often the case with polyphasic MEPs, it can get lost when averaging across multiple trials (Keil et al. 2022). Frequency domain analysis decomposes the EMG signal into oscillations of varying frequencies. The contribution of each frequency to the overall EMG signal is calculated, commonly referred to as the power spectrum of the signal (Machetanz et al. 2021a). Using a time–frequency analysis, the signal’s power can be evaluated over time, enabling analysis of both spectral and temporal information. The power spectral values cannot be negative, meaning unlike time-domain analysis, non-phase-locked features will be preserved when averaging across trials (Keil et al. 2022). Although time–frequency analysis is prevalent in neuroscience, particularly in electroencephalography studies (Wacker and Witte 2013; Herrmann et al. 2014), its adoption in a clinical setting has been relatively slow. Nevertheless, recent publications demonstrate the emerging use of time–frequency analysis in clinical applications (Singh et al. 2019; Machetanz et al. 2021a, 2021b).

A strength of this study was the use of a time–frequency analysis to detect MEPs (Tecuapetla-Trejo et al. 2021). Time–frequency analyses reduce data processing time and decrease manual inspection subjectivity (Tecuapetla-Trejo et al. 2021). Using the time–frequency approach, MEPs smaller than 50 μV can be detected objectively and are informative of a subthreshold level of activation of the CM pathway. In fact, it has been shown that responses below 10 μV can be consistently recorded (Li et al. 2022). The time–frequency analysis identifies MEPs without using an arbitrary amplitude criterion, or manual inspection which can be time-consuming and prone to subjective error.

### Limitations

The present study has some limitations. Some of the parameters used to derive the threshold matrix are necessarily arbitrary, such as the muscles selected and the steps for %MSO increments (i.e. 10%). Changing either of these may alter the composition of the threshold matrix. The full range of stimulator intensities at fixed intervals was used to capture all possible responses, irrespective of the participants’ RMT. The parameters of 10% MSO increments and ten stimulations at each intensity were chosen to ensure a tolerable experimental session duration. As expected, the area of interest for threshold matrices consists of intensities around RMT where responses transition from subliminal through subthreshold to suprathreshold. The shift from subliminal to suprathreshold responses occurs rapidly in healthy older adults, evidenced by the steep linear portion of the S–R curve. Therefore, the 10% MSO increments may not have been sensitive enough to capture subthreshold responses in healthy older adults.

The present study showed no differences in threshold matrix elements between hand and forearm muscles. Corticomotor innervation increases with more distal musculature (Chen et al. 1998), so it is reasonable to hypothesise that there would be compositional differences between the distal and proximal muscle groups. One explanation could be that the 10% MSO increments were not sensitive enough to capture the differences between muscle groups. Future studies may usefully explore smaller stimulation intensity increments specifically around RMT, if it is known or determined in advance. Smaller intensity increments may yield a more sensitive threshold matrix to explore differences between proximal versus distal muscles, for example.

A fixed testing order of TMS measures was used in this study so that any order effect would consistently influence the data collected. However, an alternative approach would have been to randomise the stimulation order between participants whilst keeping the order fixed within participants, so any order effects were consistent for the reliability measures.

### Conclusion

The present study introduced a novel TMS-derived MEP threshold matrix to examine corticomotor excitability in a cohort of neurologically healthy older adults. The threshold matrix had good or excellent reliability for suprathreshold and subliminal elements, in a sense confirming the known reliability of RMT. The relatively poor reliability of the subthreshold element of the matrix was not unexpected due to the relatively infrequent subthreshold responses in neurologically healthy older adults. Another confirmatory finding was the similarity between dominant and non-dominant threshold matrices and the absence of a relationship between the subthreshold element and RMT. These findings show that robust metrics can be derived from a threshold matrix without confounds due to differences in side/dominance or RMT. Future research might usefully explore matrix composition in sub-acute stroke patients to see how it changes over time during recovery or may be influenced by novel interventions which target the corticomotor system.

### Supplementary Information

Below is the link to the electronic supplementary material.

Supplementary file1 (DOCX 580 KB)

## Data Availability

The datasets generated during the current study are available from the corresponding author on reasonable request.

## Abbreviations

ADM: Abductor digiti minimi muscle
ALR: Amalgamation log ratios
BF: Bayes factor
ECR: Extensor carpi radialis muscle
EMG: Electromyography
FCR: Flexor carpi radialis muscle
FDI: First dorsal interosseous muscle
ICC: Intraclass correlation coefficient
M1: Primary motor cortex
MEP: Motor evoked potential
MSO: Maximum stimulator output
RMT: Resting motor threshold
S–R curve: Stimulus–response curve
TMS: Transcranial magnetic stimulation
