# Supplementary material for: Reliability of a TMS-derived threshold matrix of corticomotor function
Source: Exp Brain Res. 2023 Oct 28;241(11-12):2829–43. doi: 10.1007/s00221-023-06725-3 (PMC10635992; doi:10.1007/s00221-023-06725-3)
Supplement: Supplementary file 1 — Supplementary file1 (DOCX 580 KB) [file 221_2023_6725_MOESM1_ESM.docx]

**Supplementary Material**


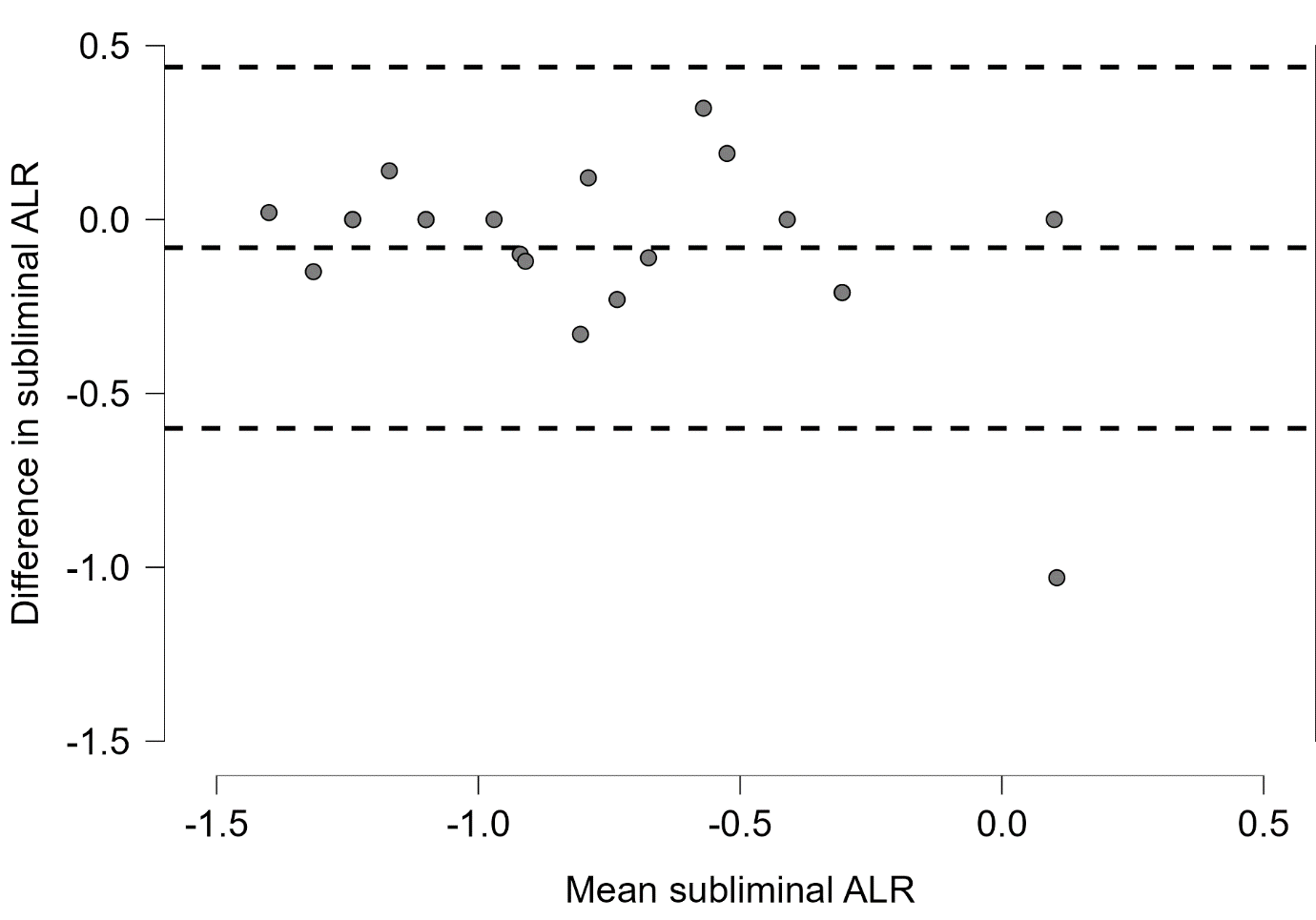


**0.44**

**-0.08**

**-0.60**

**Figure S1. Bland Altman plot for dominant subliminal responses.** Mean subliminal amalgamated log ratio (ALR) across the two experimental sessions plotted against the difference in subliminal ALR between the two experimental sessions. The mean difference of -0.08 is shown as the middle horizontal dashed line. The upper and lower 95% confidence intervals of the difference are shown as the upper (0.44) and lower (-0.60) dashed lines, respectively.


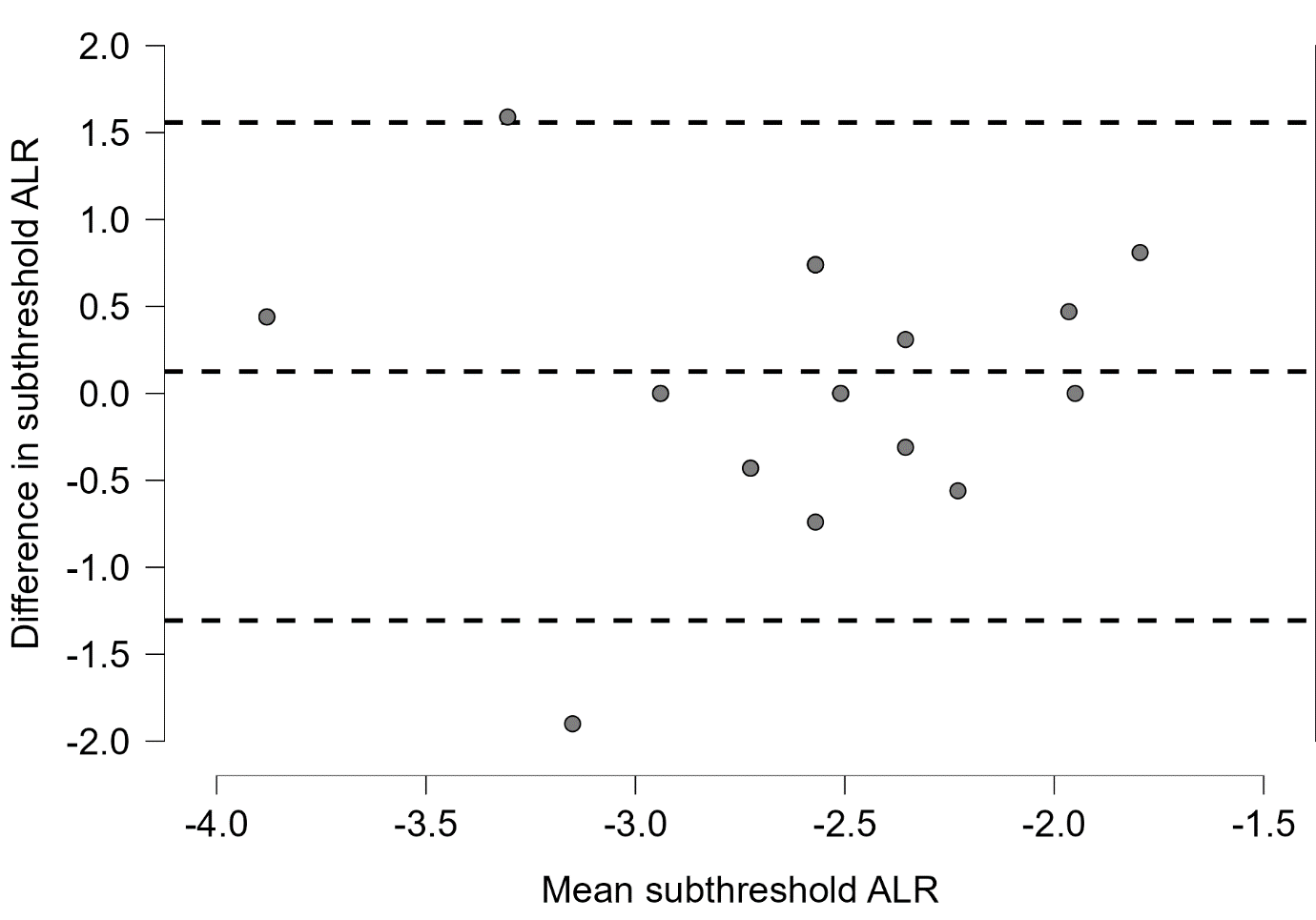


**1.56**

**0.13**

**-1.31**

**Figure S2. Bland Altman plot for dominant subthreshold responses.** Mean subthreshold amalgamated log ratio (ALR) across the two experimental sessions plotted against the difference in subthreshold ALR between the two experimental sessions. The mean difference of 0.13 is shown as the middle horizontal dashed line. The upper and lower 95% confidence intervals of the difference are shown as the upper (1.56) and lower (-1.31) dashed lines, respectively.


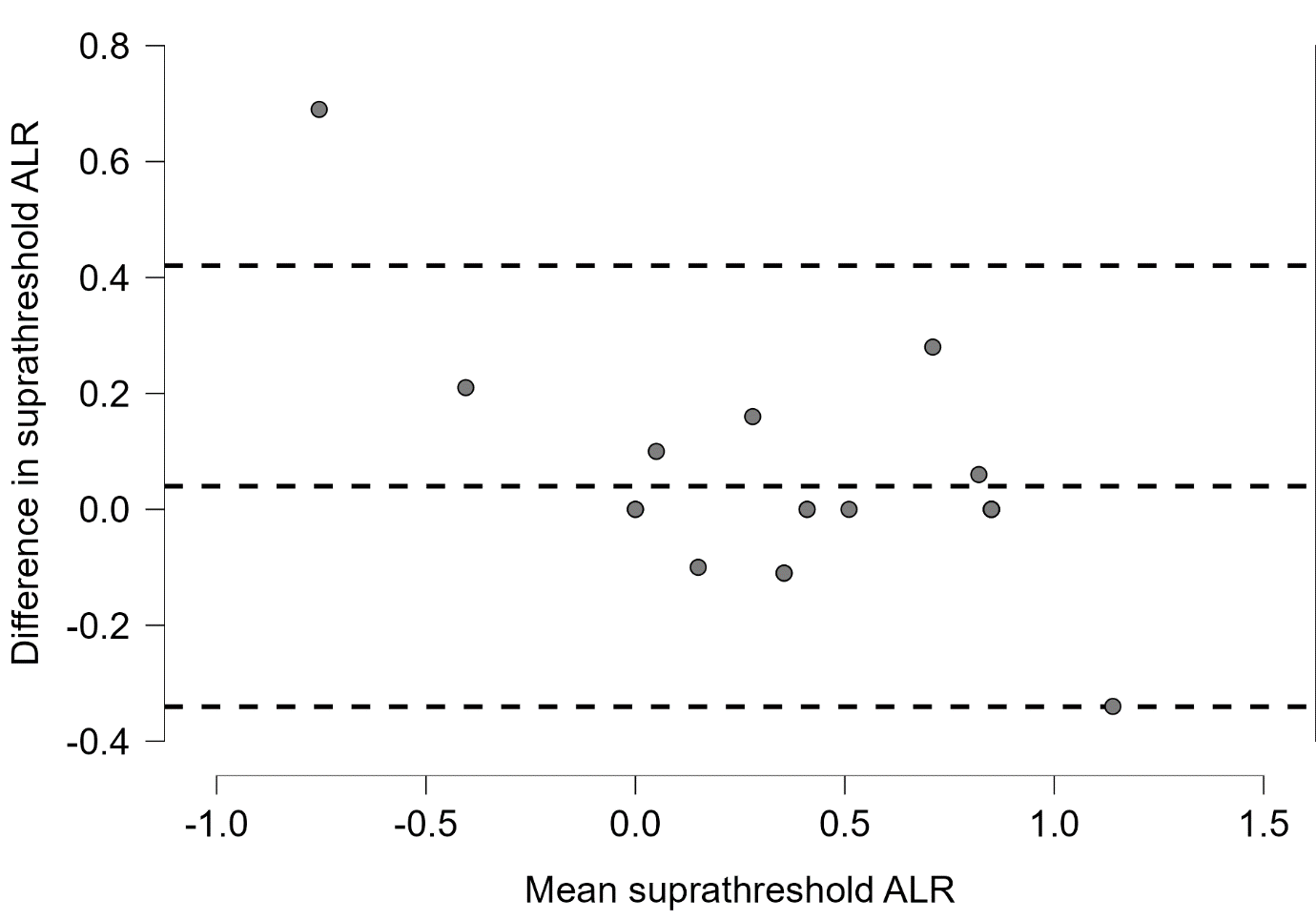


**0.42**

**0.04**

**-0.34**

**Figure S3. Bland Altman plot for dominant suprathreshold responses.** Mean suprathreshold amalgamated log ratio (ALR) across the two experimental sessions plotted against the difference in suprathreshold ALR between the two experimental sessions. The mean difference of 0.04 is shown as the middle horizontal dashed line. The upper and lower 95% confidence intervals of the difference are shown as the upper (0.42) and lower (-0.34) dashed lines, respectively.


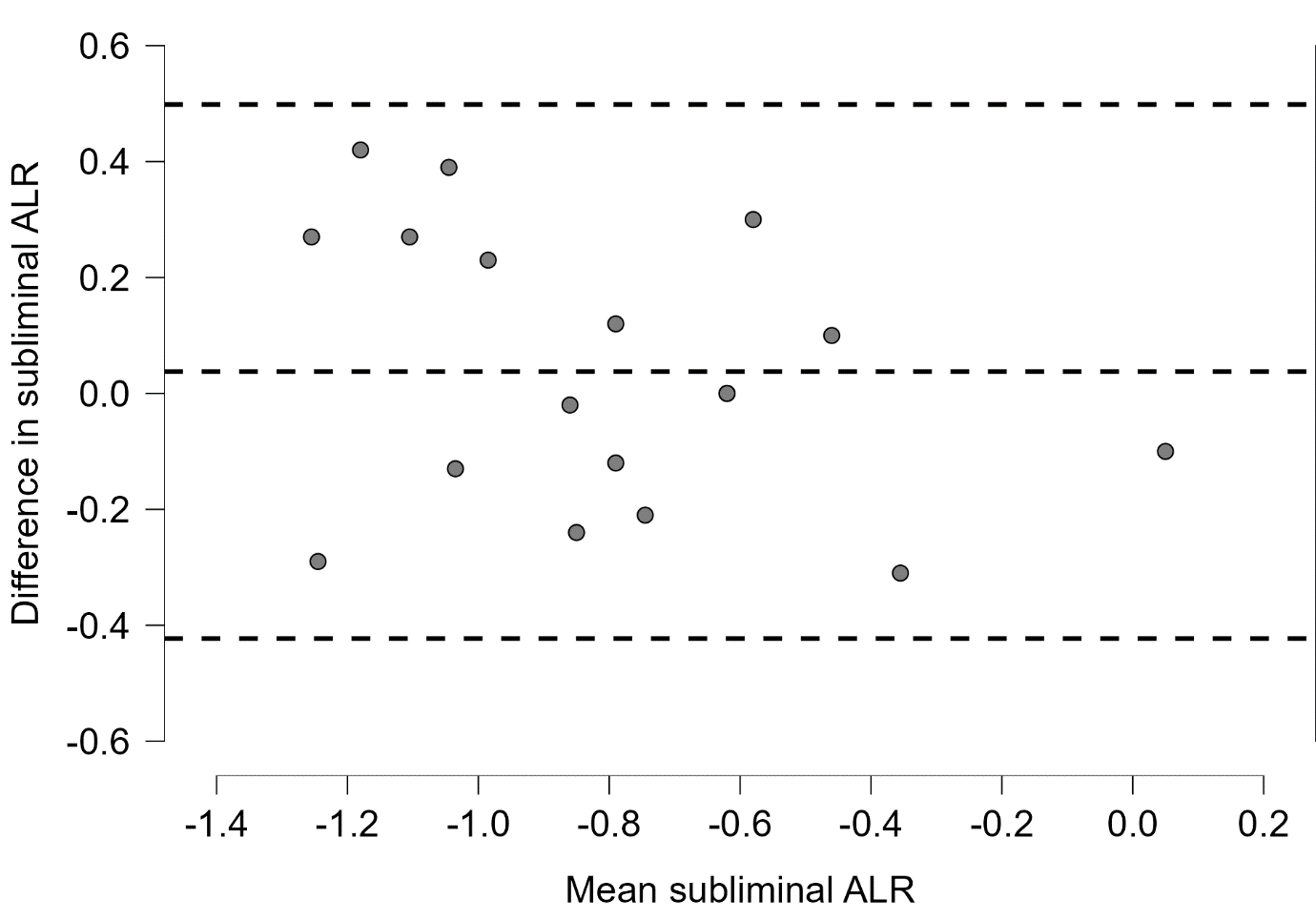


**0.50**

**0.04**

**-0.42**

**Figure S4. Bland Altman plot for non-dominant subliminal responses.** Mean subliminal amalgamated log ratio (ALR) across the two experimental sessions plotted against the difference in subliminal ALR between the two experimental sessions. The mean difference of 0.04 is shown as the middle horizontal dashed line. The upper and lower 95% confidence intervals of the difference are shown as the upper (0.50) and lower (-0.42) dashed lines, respectively.


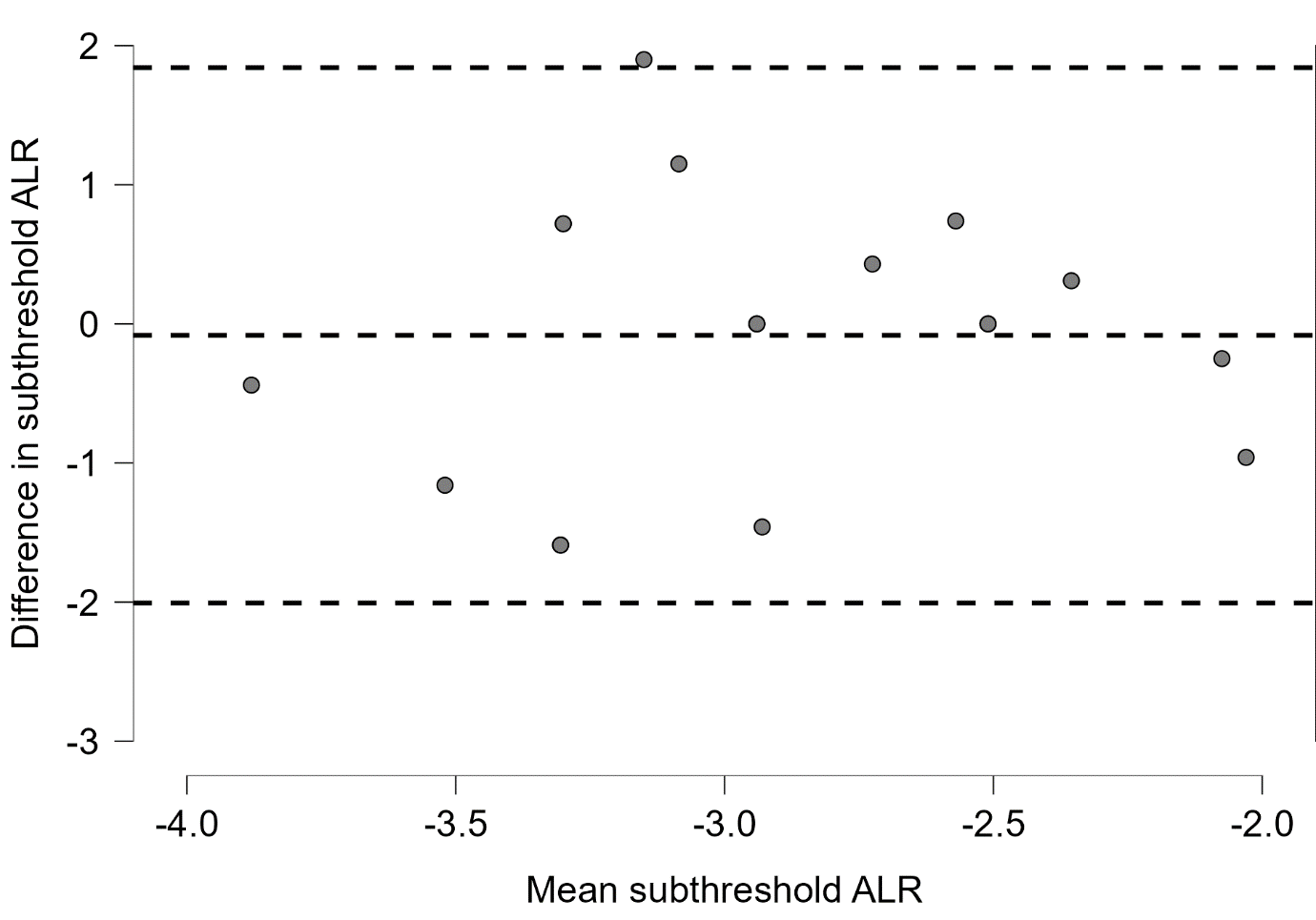


**1.84**

**-0.08**

**-2.01**

**Figure S5. Bland Altman plot for non-dominant subthreshold responses.** Mean subthreshold amalgamated log ratio (ALR) across the two experimental sessions plotted against the difference in subthreshold ALR between the two experimental sessions. The mean difference of -0.08 is shown as the middle horizontal dashed line. The upper and lower 95% confidence intervals of the difference are shown as the upper (1.84) and lower (-2.01) dashed lines, respectively.


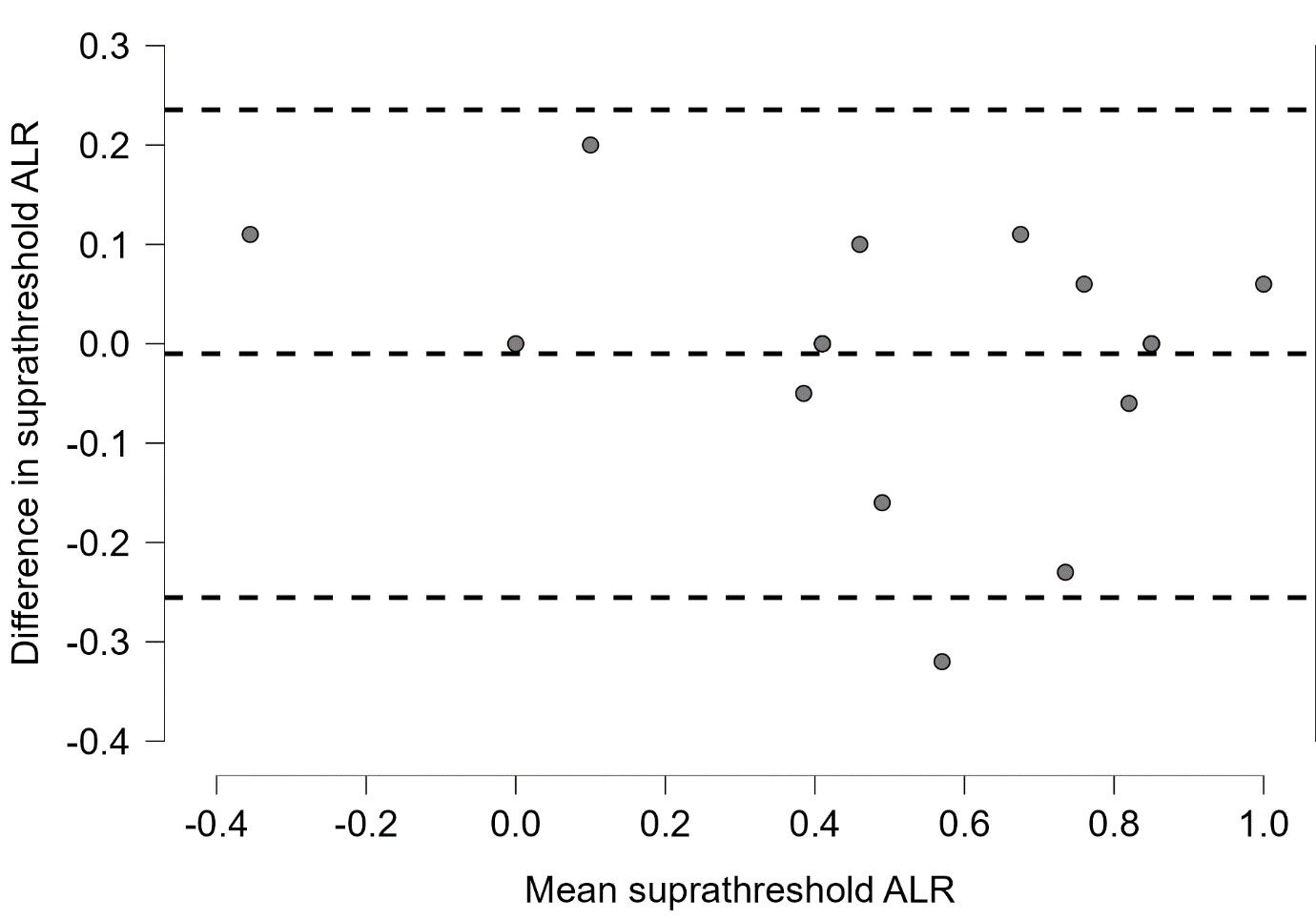


**0.24**

**-0.01**

**-0.26**

**Figure S6. Bland Altman plot for non-dominant suprathreshold responses.** Mean suprathreshold amalgamated log ratio (ALR) across the two experimental sessions plotted against the difference in suprathreshold ALR between the two experimental sessions. The mean difference of -0.01 is shown as the middle horizontal dashed line. The upper and lower 95% confidence intervals of the difference are shown as the upper (0.24) and lower (-0.26) dashed lines, respectively.
